# The protective effect of *Curcuma longa* on male infertility induced by thioacetamide

**DOI:** 10.5455/javar.2024.k828

**Published:** 2024-09-30

**Authors:** Fatima S. Alaryani, Fatima A. Jaber, Boudor S. Almutiri, Suzan B. Abdu, Arif Mohammed, Asmaa H. Al-Robiee

**Affiliations:** 1 Department of Biological Sciences, College of Science, University of Jeddah, Jeddah, Saudi Arabia; 2 Biology Department, Faculty of Science, Umm Al Qura University, Makkah, Saudi Arabia

**Keywords:** *Curcuma longa*, Thioacetamide, male infertility, oxidative stress, therapeutic effects

## Abstract

**Objective:**

This study aimed to investigate the impact of thioacetamide (TAA) on the structure and function of the testes and assess the therapeutic effects of *Curcuma longa* (Cl) against TAA-induced toxicity in rats.

**Materials and Methods:**

Thirty-two male albino rats weighing 180–200 gm and aged 11–12 weeks were randomly separated into four groups. The control group was given normal saline, the Cl group was orally administered Cl (500 mg/kg/day), the TAA group received intraperitoneal TAA (200 mg/kg body weight, three times/week), and the Cl with TAA group received Cl orally two hours before TAA administration. After 8 weeks, all rats were anesthetized, and body and testis weights were recorded. Morphological and histological assessments as well as biochemical analyses were conducted.

**Results:**

The study revealed a significant decrease in both body and testis weights in the TAA group, accompanied by a substantial increase in luteinizing hormone (LH), follicle-stimulating hormone (FSH), and malondialdehyde (MDA) levels. Testosterone (T) and glutathione (GSH) were significantly decreased in the TAA-treated group compared to the control. Conversely, the Cl-treated group exhibited a substantial decrease in LH, FSH, and MDA levels while showing a significant increase in T and GSH.

**Conclusion:**

Cl has been found to have a potential therapeutic role in mitigating TAA-induced testicular damage by acting as an antioxidant. This is supported by a significant decrease in oxidative stress markers and supporting hormonal levels. Further research is needed to understand the underlying mechanisms and explore the clinical applicability of Cl in preventing and treating testicular toxicity.

## Introduction

There is a lack of data in Saudi Arabia about infertility, which is a global issue that must be addressed in the future to prevent it from becoming a serious public health issue. Between 1990 and 2017, the prevalence rate of infertility rose by 0.291% for men and 0.370% for women annually [1]. Globally, 15% of couples who are of reproductive age struggle with infertility [2]. Sengupta et al. [3] reported that research investigating the impact of lifestyle and environmental variables on male reproductive potential has been stimulated by reports concerning the deterioration in semen quality and its likely implications on male fertility. Several psychological, medical, and societal issues have been connected to infertility [4].

A sulfur-containing substance called thioacetamide (TAA), TAA acid/acetohexamide, is frequently utilized in hospitals and industries to substitute hydrogen sulfide [5]. The hypothesis that TAA directly damages testicular tissue by inducing oxidative processes was supported by the elevated indicators of lipid peroxidation and the reduced levels of natural antioxidants in the testicular tissue [6]. TAA may affect spermatogenic cells directly or indirectly by causing harm to the Sertoli cells that sustain them. Rats with cirrhosis brought on by TAA have been shown to have reduced antioxidant enzyme activity in their testes [6,7].

The testes are equipped with a complex array of antioxidant enzymes and free radical scavengers to guarantee further that oxidative stress (OS) does not affect the organ’s dual spermatogenic and steroidogenic activities. These antioxidant defense mechanisms are critical because peroxidative damage is now thought to be the primary factor causing impaired testicular function, which is the basis for the pathological outcomes of various conditions ranging from diabetes and xenobiotic exposure to testicular torsion [8]. Oxygen radical generation that surpasses the stressed tissue’s antioxidant capability leads to OS. Various factors linked to male infertility act as inducers of OS. For instance, X-irradiation, exposure to environmental toxicants, and the physical conditions of varicocele and cryptorchidism have been proven to elevate testicular OS levels [9]. This, in turn, results in an upsurge in germ cell apoptosis, leading to subsequent hypospermatogenesis. Stressful conditions of this nature can induce alterations in the dynamics of testicular microvascular blood flow, germ cell apoptosis, and endocrine signaling [10].

Turmeric, scientifically known as *Curcuma longa* (Cl), is a perennial *Zingiberaceae* family plant extensively grown in tropical regions such as India, China, Pakistan, Kenya, Ghana, and Nigeria [11]. Its distinct yellow color is attributed to the presence of curcumin. This plant offers a range of health benefits, including anti-inflammatory, anti-carcinogenic, antioxidant, anti-diabetic, lipid-lowering, hepatoprotective, anti-obesity, anti-HIV, and immunomodulating effects [12]. Furthermore, Cl extract has been shown to reduce lipid aggregation by regulating redox processes in the endoplasmic reticulum [13]. It exhibits a diverse range of effects, including antiproliferative, antiviral, antirheumatic, anticancer, antidiabetic, antivenomous, diuretic, hypocholesterolemic, larvicidal, anti-thrombotic, antihepatotoxic, anti-diarrheal, anti-inflammatory, carminative, hypotensive, antimicrobial, antioxidant, insecticidal, and antityrosinase effects, among others [14,15]. Another phytochemical, such as *Nigella sativa* can protect the testicular damage caused by TAA in rats [6].

The present study aimed to investigate the therapeutic potential of Cl juice in addressing TAA-induced infertility in male rats. Through a comprehensive analysis of biochemical, morphological, histological, and OS parameters, we seek to uncover Cl’s protective impact on testicular tissue. Our objectives include assessing improvements in body and testis weights, hormonal levels, and reductions in OS markers following Cl administration.

## Materials and Methods

### Ethical approval

The Animal Care and Use Committee of King Abdulaziz University established ethical guidelines for experimental treatments (Reference No. 71-24). The rats were categorized into four groups, each consisting of eight, and underwent an 8-week treatment regimen.

### Animals

Thirty-two adult healthy male albino rats (Wistar) weighing 180–200 gm and 3–4 weeks old were used as an experimental model. The experimental animals were procured from the animal house of the Experimental Animal Unit at King Fahd Medical Research Center, King Abdulaziz University, Jeddah, Saudi Arabia. These animals were maintained in a controlled environment at a constant temperature of 25°C ± 2°C, with relative humidity ranging from 50% to 60%, and subjected to a 12-h light-dark cycle. They were provided with free access to a standard diet and water.

### Chemicals

TAA was purchased from (Sigma Aldrich Crop. St. Louis, MO, USA; Lot#. BCCB7885). TAA was dissolved in 16.3 gm/100 ml of distilled water. Cl Pure Cl powder (product code: 091030) was purchased from the local market in its natural and fresh form. It was cleaned and ground with black pepper, kept in a tightly closed box to prevent the entry of external influences, and placed in a cool place. The method was repeated weekly and given to the rats orally.

### Experimental design

Thirty-two male albino rats (Wistar) with body weights of 180–200 gm were randomly assigned to four equal groups, as follows:

*Control group:* normal saline solution orally (G1).

*Cl group:* rats were orally administered daily with Cl (500 mg/kg/day; G2).

*TAA group* (*n = *8 rats): rats were intraperitoneally administered (200 mg/kg body weight, three times/week; G3).

*Cl with TAA group:* rats were orally administered Cl (daily), two hours earlier before TAA administration (three times/week; G4).

### Body weight and sample collection

Each group’s body weight was measured weekly. Following the last dose of the study, animals were subjected to a 12-h fasting period during which they were not allowed to eat. Subsequently, the animals were anesthetized, and blood samples were collected from the orbital vein. The collected blood samples were centrifuged at 3,000 rpm and 4°C for 10 min using SIGMA_22003. For biochemical analysis, the serum obtained was stored at −20°C. Animals were killed, and their testes were removed and weighed after being rinsed in cold saline. For the histological study, portions of testis were fixed in four percent formaldehyde in 0.1 M sodium phosphate buffer (pH 7.4) overnight at 4°C. The remaining tissue was kept at −20°C until it was time to be tested.

### Biochemical analysis

Using an enzyme-linked immunosorbent assay (ELISA) kit (Cat No. MBS764675), the levels of luteinizing hormone (LH), the ELISA kit (Cat No. MBS2502190), the levels of follicle-stimulating hormone (FSH), and the ELISA kit (Cat No. MBS9424769), the levels of testosterone (T).

### OS and antioxidant marker assessment

Following Sigma’s manufacturer’s recommendations, thiobarbituric acid was used to assess the amounts of glutathione (GSH) and malondialdehyde (MDA) in the homogenate of testis tissue from all research groups as a measure for lipid peroxidation. To the samples/standard (100 l), a ready-to-use sodium dodecyl sulfate (SDS) solution was added. The color reagent was then added to the mixture in 4 ml increments. For 1 h, the samples and standard solution vials were 26 immersed in boiling water. The reaction was halted after 10 min of incubation in an ice bath. Each vial was centrifuged for 10 min at 4°C at 1,600 × *g*. The samples and standard each received 100 μl of ready-to-use SDS solution added to them. The mixture was then mixed with 4 ml of the color reagent. Vials containing samples and standard solutions were submerged in boiling water for 1 h. The process was halted after 10 min of incubation in a cold bath. After that, each vial was centrifuged for 10 min at 4°C at 1,600 × *g*. A spectrophotometer was used to measure the absorbance of duplicated samples or standards in a 96-well plate at 532 nm. The liver GSH assay was carried out in 0.1M sodium phosphate and 0.005M ethylenediaminetetraacetic acid buffer (pH 8.0), and it was kept on ice until needed. An ELISA kit (enzyme-linked immunosorbent assay) (Cat No. MBS744364) was used in a specialized device, DSX Best 2000.

### Statistical analysis

One-way analysis of variance was used for statistical analysis (ANOVA). All calculations and analyses were done using the SPSS program. Data were expressed as means ± standard error (means ± SE). Differences were deemed statistically significant at *p < *0.05.

## Results

### Morphological results

In all experimental groups, we found a significant decrease in the final body weight in the TAA group compared to the control group (*p < *0.05). However, there was a slight increase in the final body weight of animals in the Cl group in the eighth week compared to the control group (Table 1). Regarding testicle weight, there was a significant decrease among all experimental groups compared to the control group (*p < *0.05) (Table 1).

### Hormone profile

**Figure 1.**
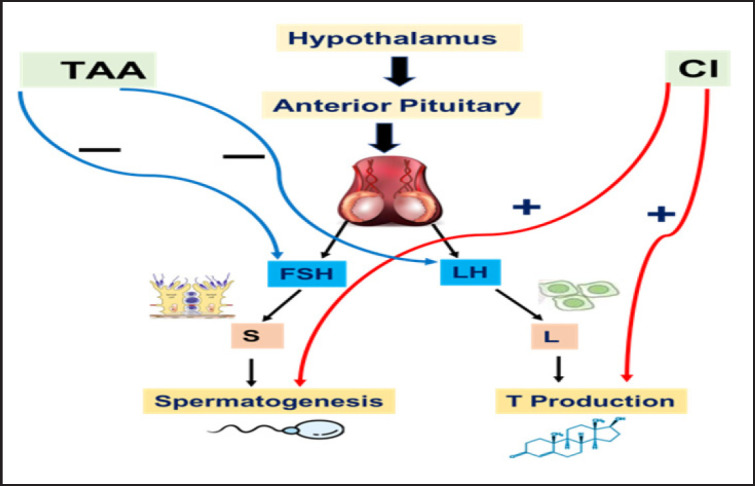
The potential effect of TAA on hormonal profile in male rats and therapeutic role of CI in mitigating TAA-induced hormone imbalance.

When comparing the TAA and Cl groups to the control group, there was a substantial decrease in T (*p < *0.05) but a significant increase in LH and FSH (Table 2). The mode of action of TAA affects hormone production and is presented in Figure 1.

**Table 1.** Comparison of mean value of final body weights and testicle weights among the studies groups by analysis of variance and least significant difference tests.

Variables	G1	G2	G3	G4
Body weight (week)				
First	196.17 ± 2.53	174.00 ± 0.85	191.88 ± 1.26	183.63 ± 1.66**
Second	237.17 ± 3.82	217.13 ± 2.16	200.94 ± 2.46*	192.63 ± 2.51
Third	267.75 ± 5.37	251.75 ± 5.22	218.13 ± 4.18*	209.63 ± 6.22
Fourth	283.00 ± 6.91	280.63 ± 6.67	236.88 ± 4.64*	236.75 ± 9.03
Fifth	303.25 ± 5.75	302.75 ± 8.52	236.00 ± 3.16*	247.88 ± 9.15
Sixth	327.92 ± 4.86	313.13 ± 9.00	249.94 ± 5.55*	254.13 ± 10.02
Seventh	349.58 ± 5.58	339.88 ± 9.90	264.44 ± 5.88*	271.88 ± 11.22
Eighth	342.42 ± 5.15	360.50 ± 9.64	268.69 ± 5.03*	272.38 ± 10.13
The ratio of total increase in body weight (%)	74.55%	107.18%	40.03%	48.33%
Testicular weight (gm)	1.18 ± 0.03	1.11 ± 0.06	0.94 ± 0.04*	0.84 ± 0.05

*,** Significance versus G1. G1: rats were given normal saline solution orally. G2: rats were orally administered daily with Cl (500 mg/kg/day). G3; rats were intraperitoneally administered (200 mg/kg body weight, three times/week). G4; rats were orally administered Cl (daily), 2 hours earlier before TAA administration (three times/week). Data were expressed as mean+/− standard error of the mean; significance among groups was made using the One-Way ANOVA test followed by least significant difference (LSD).

**Table 2.** Comparison of mean value of biochemical measured parameters among the studies groups.

Variables	G1	G2	G3	G4
Testosterone (ng/dl)	344.0 ± 24.52	315.60 ± 10.54	214.14 ± 42.37*	333.29 ± 37.49**
LH (mIU/ml)	4.24 ± 0.53	3.99 ± 0.36	8.90 ± 1.16*	5.14 ± 0.93**
FSH (mIU/ml)	4.00 ± 0.32	3.87 ± 0.34	8.05 ± 1.04*	4.78 ± 0.78**

*,**: Significance versus G1. G1: rats ere given normal saline solution orally. G2: rats were orally administered daily with Cl (500 mg/kg/day). G3; rats were intraperitoneally administered (200 mg/kg body weight, three times/week). G4; rats were orally administered Cl (daily), 2 hours earlier before TAA administration (three times/week).

Data were expressed as mean+/- standard error of the mean; significance among groups was made using the One-Way ANOVA test followed by least significant difference (LSD).

### Oxidative and antioxidant markers

**Figure 2.**
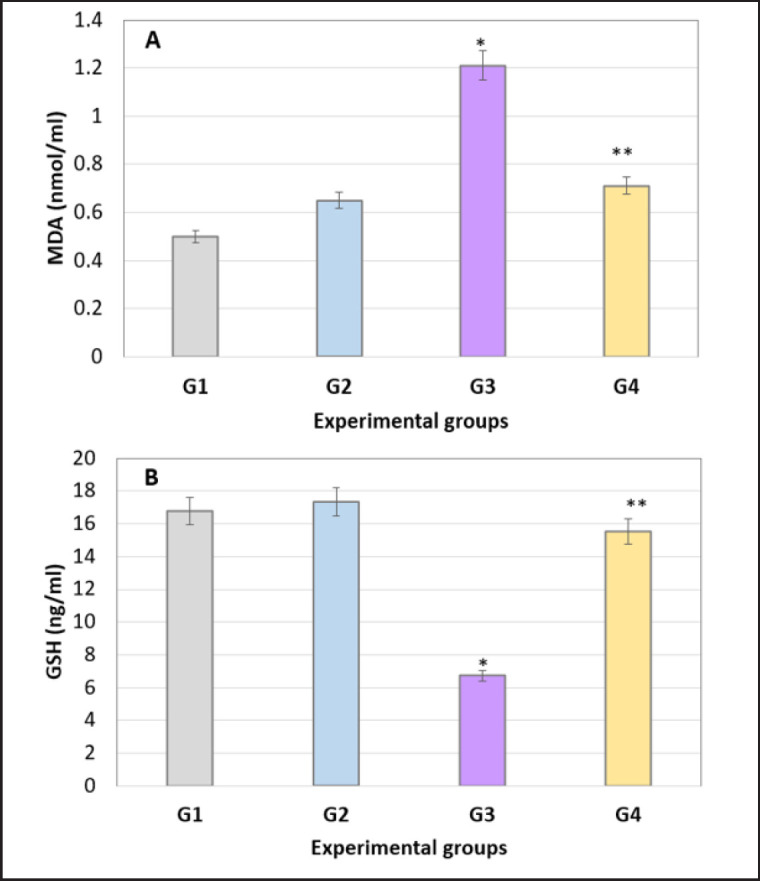
A, Comparison of Testis tissue homogenate levels of MDA (nmol/ml) among studied groups. B, Comparison of Testis tissue homogenate levels of GSH (ng/ml) among studied groups. Data were expressed as mean+/− standard error of the mean; significance among groups was made using the One-Way ANOVA test followed by least significant difference (LSD). *: Significance versus G1. G1: rats were given normal saline solution orally. G2: rats were orally administered daily with Cl (500 mg/kg/day). G3; rats were intraperitoneally administered (200 mg/kg body weight, three times/week). G4; rats were orally administered Cl (daily), 2 hours earlier before TAA administration (three times/week).

Testis tissue was prepared to estimate the level of MDA in all experimental groups. In comparison to the control group, the testis of the rat from TAA and Cl with TAA showed a significant increase (*p < *0.05) in MDA levels and a significant decrease (*p < *0.05) in GSH levels (Figs. 2A and B).

### Histological examinations

**Figure 3.**
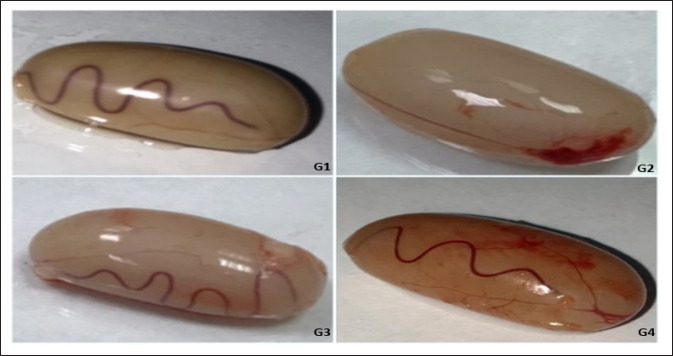
Morphology of the testicle, G1, G2, G3 showing expansion and appearance of spots on the outer surface, G4 showing vasodilation. G1: rats were given normal saline solution orally. G2: rats were orally administered daily with Cl (500 mg/kg/day). G3; rats were intraperitoneally administered (200 mg/kg body weight, three times/week). G4; rats were orally administered Cl (daily), 2 hours earlier before TAA administration (three times/week).

**Figure 4.**
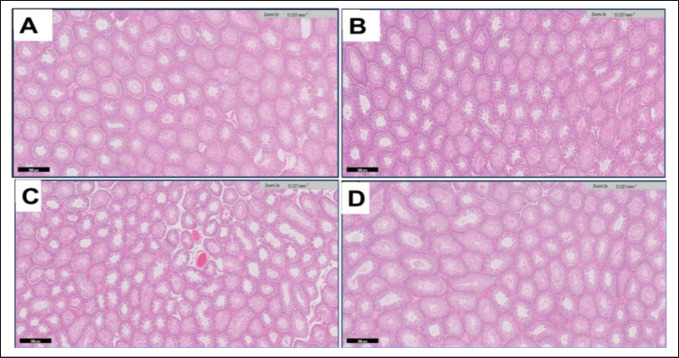
(A-D) A light microscopic micrograph depicting the testicular tissue from adult rats in G1 (Fig. 4A) and G2 (Fig. 4B). The image illustrates a healthy and dense distribution of seminiferous tubules. Notably, the seminiferous tubules display diverse geometric profiles, appearing in shapes that range from rounded to oval. G3 (Fig. 4C) shows a low density of seminiferous tubules and is present in an irregular shape. Slight interstitial edema between the seminiferous tubules and congestion of blood vessels. G4 (Fig. 4D) shows the normal density of seminiferous tubules with various shapes (rounded, oval, or irregular), H&E staining. Scale bar = 500 μm.

**Figure 5.**
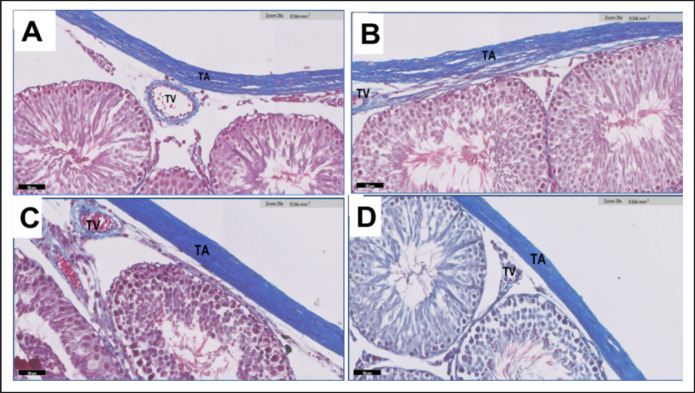
(A-D) Photomicrographs of sections in the testis of adult male rats G1 (Fig. 5A) & G2 (Fig. 5B) showing the testicular capsule formed of Tunica albuginea (TA) and Tunica vasculosea (TV). T. albuginea is formed of several layers of collagen fibers with spindle-shaped cells. A small blood vessel in T. vasculosea. Few collagen fibers surround each seminiferous tubule. G3 (Fig. 5C) shows an increase in collagen fibers in the TA. There were dilated and congested blood vessels within TV. G4 (Fig. 5D) shows TA contains oval, deeply stained nuclei. T. vasculosea contains slightly congested blood vessels. Masson’s trichrome staining. Scale bar = 50 μm.

**Figure 6.**
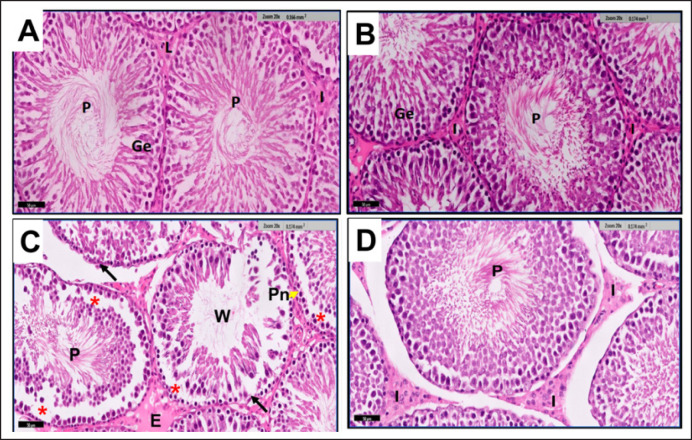
(A-D) Microscopic examination of testicular tissue from adult rats in G1 (Fig. 6A) and G2 (Fig. 6B) reveals well-formed seminiferous tubules surrounded by the Basal Lamina (BL) and featuring a normal lining of stratified Germinal epithelium (Ge). Lumina is observed to contain aggregations of sperms (P), while narrow interstitial spaces (I) exhibit clusters of L. In contrast, G3 (Fig. 6C) exhibits seminiferous tubules with an apparent reduction in layers of germinal epithelium. Some tubules rest on an irregular basement membrane (black arrow), displaying sloughing of germ cell layers (*) and pyknotic nuclei (Pn) of spermatogonia, along with wider lumina (W) and edema (E) in interstitial spaces. G4 (Fig. 6D) displays seminiferous tubules with mostly regular contours, stratified germinal epithelium, and P. I are relatively narrow, housing clusters of cells. The figures were obtained through H&E staining. Scale bar = 20 μm.

**Figure 7.**
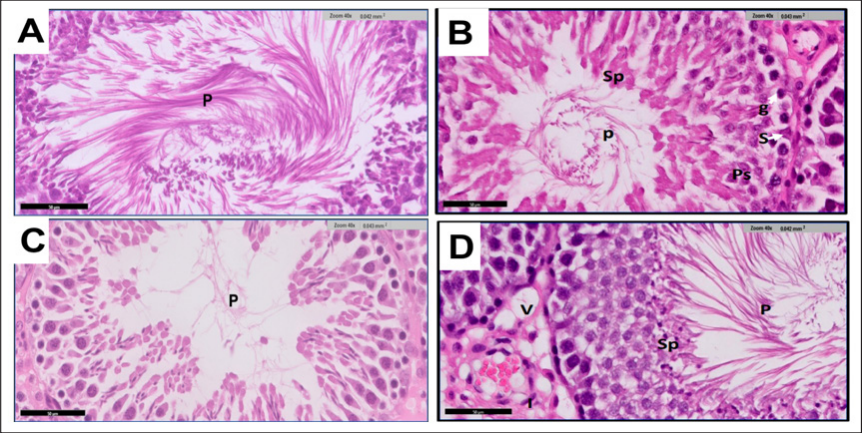
(A-D) A light microscopic micrograph of testicular tissue from adult rats in G1 (Fig. 7A) depicts normal seminiferous tubules, sheathed with the Basal Lamina (BL) and featuring a normal lining of stratified Germinal epithelium (Ge). Lumina contain aggregations of sperms (P), and narrow interstitial spaces (I) reveal clusters of Leydig cells (L). In G2 (Fig. 7B), the arrangement of stratified germinal epithelium showcases various types of spermatogenic cells, including resting Spermatogonia (g) with small, rounded nuclei, Primary Spermatocytes (Ps) with large, rounded nuclei, and small, rounded Spermatids (Sp). Sertoli cells (S) are interspersed among spermatogenic cells, while sperm agglutination is observed in the tubule center. G3 (Fig. 7C), exhibits a lower density of sperm P compared to the control group. G4 (Fig. 7D), displays nearly regular seminiferous tubules containing sperm aggregations P, small, rounded Spermatids (Sp), and Sertoli cells (S). Vacuolation (V) is evident in the interstitial tissue (I) and some germ cells. The figures were obtained through H&E staining; the scale bar is 20 μm.

**Figure 8.**
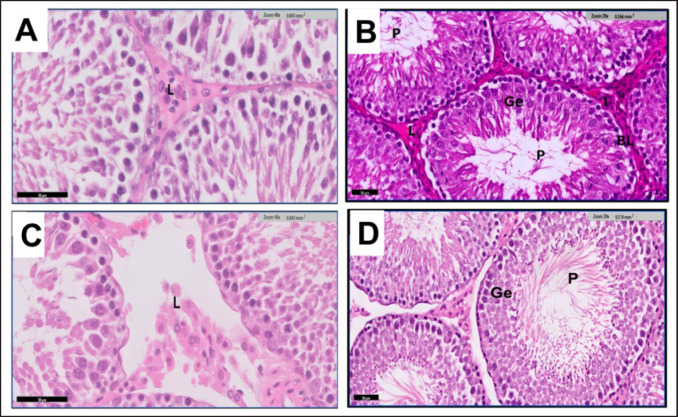
(A-D) Light microscopic testicular tissue from adult rats in G1 (Fig. 8A) shows the interstitial cells of Leydig cells (L) are present in large groups between interstitial tissues. The cells have an acidophilic cytoplasm and rounded or oval, deeply stained nuclei. G2 (Fig. 8B) shows a normal appearance of seminiferous tubules with stratified germinal epithelium cells (Ge) resting on regular basal lamina (BL). Tubules appear filled with sperms (P) in the Lumina. Normal interstitial spaces (I) with clusters of L. G3 (Fig. 8C) shows degeneration and deformation of Leydig’s cells. G4 (Fig. 8D) shows restoration of the testicular structure in most seminiferous tubules lined by stratified Ge and aggregations of sperms P in the center tubule. However, some seminiferous tubules have shrunken basal lamina around them. Interstitial spaces are relatively arranged compared with the control group. H&E staining. Scale bar = 50 μm.

By examining the cross sections of the testis in the G4 after the eighth week of the experiment, we found a decrease in the size of seminiferous tubules compared to the control group. The seminiferous tubules have low density and are found in many shapes. They are either oval, circular, or irregularly shaped. In addition to edema between the seminiferous tubules and congestion of blood vessels (Fig. 3). Increase of collagen fibers in the Tunica Albuginea with dilation and congesting of blood vassal within Tunica Vacuoles in G3 (Fig. 4). Germ cells were seen to be sparsely populated in some of the seminal vesicles. Supporting cells are separated from the basal lamina and have necrosis cells. Also, the pyknotic nuclei of spermatogonia are wide lumens. They are dark pigmented, and the cells are lysed and damage spermatogonia. Primary spermatocytes are round and low in density compared to the control groups. They are lysed and have lost their cytoplasm or have dark nuclei. Secondary spermatocytes are absent (Fig. 5). The seminiferous tubules are less dense in terms of the content of sperm cells (Fig. 6). Leydig’s cells are decomposed and deformed in clusters between the seminal vesicles. Sertoli cells are found among the germ cells lying on the basement membrane and are pale in color (Figs. 7 and 8).

## Discussion

In all experimental groups, we found a considerable reduction in the final body and testicular weight in the TAA group compared to the control group. However, there was a slight increase in the final body weight of animals in the Cl group in the eighth week, compared with the control. The obtained results may be due to the toxic effect of TAA on the animal body. The study’s results suggest that Cl improves testicular morphology and ameliorates TAA-induced toxicity, which is consistent with [16].

The present investigation revealed a notable increase in LH and FSH; however, there was a significant decrease in T compared to the control group. Notably, FSH emerged as the most distinguishing hormone for classifying infertility, showcasing the highest diagnostic value in G3. Our findings align with those of Memarzia et al. [17], who observed a decline in serum T levels due to TAA toxicity. This rise in FSH and LH levels corresponds to Koblihová et al.’s [18] findings, emphasizing the utility of increased FSH and LH in managing male infertility. FSH is recognized as a dependable indicator of germinal epithelial damage and has been associated with conditions like azoospermia and severe infertility. Remarkably, in our study, the levels of hormones treated with Cl returned to normal [19].

Moreover, the results confirm the finding of the study carried out by [20] that Cl raised T levels. The present study showed a notable rise in MDA levels and a substantial decrease in GSH levels observed in G3 and G4. The observed results could be attributed to the toxic effects of TAA and the concurrent boost in the activity of numerous antioxidant enzymes facilitated by Cl. As per our data, the administration of curcumin mitigates the OS and antioxidant imbalance induced by TAA in rats [21].

Moreover, the results confirmed the study’s findings [22]. They documented that due to their high content of polyunsaturated fatty acids in their cytoplasm and plasma membrane, as well as their weak DNA repair mechanism and antioxidant capability, sperm cells are susceptible to reactive oxygen species (ROS) [23].

Our findings are consistent with [24], who reported that the OS contributes to an elevation in germ cell apoptosis, leading to subsequent hypospermatogenesis. These stressful conditions can induce alterations in the dynamics of testicular microvascular blood flow, endocrine signaling, and germ cell apoptosis. Additionally, OS has been established as a significant factor in male infertility [9]. Moreover, ROS’s TAA-induced lipid peroxidation production disturbs the antioxidant defense system and causes a significant increase in MDA. This will cause long-lasting cell damage and a reduction in the antioxidant defense system, as observed and measured by low GSH levels [25]. Comparable, in a recent study, it was observed that the administration of curcumin to TAA-intoxicated rats substantially decreased liver damage by improving antioxidant status, inflammatory indicators, and OS levels [26].

Curcumin enhances its antioxidant capabilities by scavenging various ROS, including superoxide radicals, hydrogen peroxide, and nitric oxide radicals, and inhibiting lipid peroxidation [27]. Additionally, curcumin elevates the levels of GSH by upregulating GSH transferase and their corresponding mRNAs while inhibiting enzymes responsible for generating ROS [28]. This study found that some seminiferous tubules are irregular in shape and change in thickness from one part to another, and the seminiferous tubules are less dense in terms of the content of sperm cells. Supporting cells have low density and have lost their regularity on the basement membrane, and they have a dark and irregular nucleus. Germ cells were seen to be sparsely populated in some of the seminal vesicles. They are dark-pigmented, and the cells are lysed. Primary spermatocytes are round in shape and low in density compared to the control groups. They are lysed and have lost their cytoplasm or have dark nuclei. Secondary spermatocytes are absent. Seminiferous tubules are small in size, and supporting cells are separated from the basal lamina and have necrosis cells. The lumen of the seminiferous tubules is absent in most of the tubules. The study data indicate that an extract of Cl (150 or 450 mg/kg body weight) may help alleviate symptoms of kidney deficiency and boost T production by enhancing the steroidogenic pathway [29]. This research highlights CL as a promising nutraceutical for treating T deficiency conditions.

The interstitial spaces are wide and congested. They disrupted germinal layers and spermatogenic damage. Sertoli cells are found among the germ cells lying on the basement membrane and are pale in color. The extensive interstitial tissue shows cellular infiltrates, and Leydig’s cells are in clusters between the seminal vesicles, decomposed and deformed. The results indicating the impact of TAA showed pronounced atrophy and degeneration of testicular tubules, aligning with the findings reported by El-Demerdash et al. [6]. A recent study has clarified that curcumin, the active compound of Cl, has antioxidant properties. This feature can help mitigate the reproductive toxicity induced by heavy metals such as arsenic in rats [30].

This observation concurs with the results reported by [21,31,32], who noted the impact of TAA on spermatogenic cells or its detrimental effects on their supporting Sertoli cells. Our findings also resonate with [14,29,32], who reported that curcumin improved spermatogenic damage T levels and induced antioxidant defense.

## Conclusion

This study successfully demonstrated the detrimental impact of TAA on testicular tissue, affirming its toxic effects while concurrently revealing the protective and therapeutic efficacy of Cl. The investigation, encompassing biochemical, morphological, histological, and OS analyses, portrayed TAA-induced toxicity in male albino rats. Cl, administered concurrently, mitigated the adverse effects, notably improving body and testis weights, hormonal levels, and OS markers. The protective influence of Cl was evident in the reduction of the LH, FSH, and MDA levels, coupled with an increase in T and GSH. These findings underscore the antioxidant potential of Cl and suggest its promising role in therapeutically addressing TAA-induced testicular damage. Further research is warranted to unravel underlying mechanisms and explore clinical applications.

## List of abbreviations

Cl, *Curcuma longa; *FSH, Follicle-stimulating hormone; GSH, Glutathione; LH, Luteinizing hormone; MDA, Malondialdehyde; NO, nitric oxide; OS, oxidative stress; ROS, Reactive oxygen species; SDS, sodium dodecyl sulfate; TAA, Thioacetamide; CH_3_CSNH_2_, Thioacetimidic acid/acetothioamide; T, Testosterone.
